# Predicting ischemic stroke risk from atrial fibrillation based on multi-spectral fundus images using deep learning

**DOI:** 10.3389/fcvm.2023.1185890

**Published:** 2023-08-01

**Authors:** Hui Li, Mengdi Gao, Haiqing Song, Xiao Wu, Gang Li, Yiwei Cui, Yang Li, Zhaoheng Xie, Qiushi Ren, Haitao Zhang

**Affiliations:** ^1^ Department of Biomedical Engineering, College of Future Technology, Peking University, Beijing, China; ^2^ Institute of Biomedical Engineering, Peking University Shenzhen Graduate School, Shenzhen, China; ^3^ Shenzhen Bay Laboratory, Institute of Biomedical Engineering, Shenzhen, China; ^4^ National Biomedical Imaging Center, Peking University, Beijing, China; ^5^ Institute of Medical Technology, Peking University Health Science Center, Peking University, Beijing, China; ^6^ Department of Neurology, Xuanwu Hospital, Capital Medical University, Beijing, China; ^7^ Department of Cardiology, Beijing Yanhua Hospital, Beijing, China; ^8^ Cardio-Metabolic Medicine Center, Fuwai Hospital, Chinese Academy of Medical Sciences & Peking Union Medical College, Beijing, China

**Keywords:** ischemic stroke, atrial fibrillation, deep learning, fundus image, multi-spectrum

## Abstract

**Background:**

Ischemic stroke (IS) is one of the most common serious secondary diseases of atrial fibrillation (AF) within 1 year after its occurrence, both of which have manifestations of ischemia and hypoxia of the small vessels in the early phase of the condition. The fundus is a collection of capillaries, while the retina responds differently to light of different wavelengths. Predicting the risk of IS occurring secondary to AF, based on subtle differences in fundus images of different wavelengths, is yet to be explored. This study was conducted to predict the risk of IS occurring secondary to AF based on multi-spectrum fundus images using deep learning.

**Methods:**

A total of 150 AF participants without suffering from IS within 1 year after discharge and 100 IS participants with persistent arrhythmia symptoms or a history of AF diagnosis in the last year (defined as patients who would develop IS within 1 year after AF, based on fundus pathological manifestations generally prior to symptoms of the brain) were recruited. Fundus images at 548, 605, and 810 nm wavelengths were collected. Three classical deep neural network (DNN) models (Inception V3, ResNet50, SE50) were trained. Sociodemographic and selected routine clinical data were obtained.

**Results:**

The accuracy of all DNNs with the single-spectral or multi-spectral combination images at the three wavelengths as input reached above 78%. The IS detection performance of DNNs with 605 nm spectral images as input was relatively more stable than with the other wavelengths. The multi-spectral combination models acquired a higher area under the curve (AUC) scores than the single-spectral models.

**Conclusions:**

The probability of IS secondary to AF could be predicted based on multi-spectrum fundus images using deep learning, and combinations of multi-spectrum images improved the performance of DNNs. Acquiring different spectral fundus images is advantageous for the early prevention of cardiovascular and cerebrovascular diseases. The method in this study is a beneficial preliminary and initiative exploration for diseases that are difficult to predict the onset time such as IS.

## 1. Introduction

Cardiovascular diseases are the leading cause of mortality and disability worldwide, accounting for 32% of all death, and a major cause of rising healthcare costs (1). Of the cardiovascular diseases, atrial fibrillation (AF) is the most general type of cardiac arrhythmia. AF makes the rapid contractions of the heart weaker than normal contractions, resulting in a slower flow of blood in the atrium and further the formation of blood clots. When a clot leaves the heart, travels to the brain, and blocks blood flow through cerebral arteries, an ischemic stroke (IS) may occur (2). IS accounts for about 80% of stroke cases worldwide, while stroke have become a leading cause of morbidity and mortality worldwide (3).

AF is a major and independent risk factor for IS, making it a common and serious secondary disease of AF, and carries a fivefold increased risk of stroke (4). AF-related stroke cases are more severe than other types of stroke (5). Approximately 15%–30% of patients with AF are asymptomatic, and symptoms such as impaired functions may not be directly associated with the onset or recurrences of arrhythmia (6). Although the cause of approximately one-third of IS occurrences is unknown, silent paroxysmal AF is the most presumed etiology, especially in middle-aged healthy individuals, and it is predominantly the cause of IS rather than the trigger (7). It is reported that 61% of AF patients have IS within 1 year of their AF diagnoses, but only 13.6% received the most common warfarin therapy within 30 days of diagnosis (4). Therefore, the prevention of stroke secondary to AF can significantly reduce the rate of disability and mortality.

The general manifestations of the development of IS are blood vessel ischemia and hypoxia (8), as well as decreased vessel density and reduced oxygen metabolism in the retina (8–10). These eye-related symptoms are due to the anatomic and developmental characteristics of the eye, which is an extension of the central nervous system (CNS) (11, 12). These shared anatomical and physiological features make the eyes a good target for brain research. Since the vasculatures of the eye and the heart are exposed to the same intrinsic and environmental factors, various features in the retina may reflect the systemic health of the cardiovascular system as well as the associated risks (13, 14). Markers of cardiovascular diseases are also manifested in the eye, such as hypertensive retinopathy and cholesterol embolism (14). As a common cardiovascular disease, AF decreases cardiac output by 20%–30%. Blood supply is also reduced along the CNS, causing ischemia and hypoxia of the entire CNS including the retina (15, 16) and the reduced retinal blood flow (17). Hence, the fundus involved to blood oxygen and blood flow is an important window for studying AF and AF secondary IS.

Technological advancements have led to the non-invasive visualization of blood vessels and imaging of the fundus (14). However, there is a rich collection of capillaries in the fundus (13), and microvasculature and macrovasculature are mutually affected in an intertwined manner (18). Machine learning, especially deep learning, can help capture the subtle differences in image information to identify abnormalities. Deep learning has also been leveraged for a variety of classification and prediction tasks (19). For example, deep learning combined with retinal images has been studied in the diagnosis of cardiovascular conditions (20) and stroke (21). Further, taking into consideration, the retina responds differently to lights at different wavelengths since it contains the protein photopsin in modified conformations to enable activation by light at different wavelengths (22). Therefore, in the present study, three representative deep neural networks (DNNs), Inception V3 (23), ResNet50 (24), and SE50 (25), based on fundus images at different wavelengths, were used to predict the occurrence risk of IS secondary to AF.

## 2. Materials and methods

### 2.1. Participants

This prospective study was conducted in Beijing, with a total of 150 AF and 100 IS recruited participants. The inclusion criteria of AF participants were as follows: (1) Chinese Han population, (2) aged 45–85 years old, (3) diagnosed with AF, (4) without a history of IS or symptoms of dizziness and headache, and (5) with no stroke at least for 1 year after the occurrence of AF (information obtained from the subsequent follow-up). The exclusion criteria were as follows: (1) neuropsychiatric disorders, (2) ophthalmic diseases, especially fundus diseases, and other diseases obviously affecting the eyes, and (3) any other serious physical illnesses or injuries.

The inclusion criteria of IS participants included the following: (1) Chinese Han population, (2) aged 45–85 years old, and (3) diagnosed with IS with an unknown cause or with a history of the diagnosis of AF/symptoms of persistent palpitations and arrhythmia in the last year. The exclusion criteria included the following: (1) neuropsychiatric disorders and other neurological diseases; (2) ophthalmic diseases, especially fundus diseases, and other diseases obviously affecting the eyes; and (3) other serious physical illnesses or injuries.

Sociodemographic data, including age, years of education, and current body mass index (BMI), were collected. Clinical data, including a history of substance abuse and dependence, were obtained according to medical records and self-reports and confirmed by the next of kin and family members.

The present study was approved by the ethics committee of Beijing Yanhua Hospital and Beijing Xuanwu Hospital of Capital Medical University and performed in accordance with the Declaration of Helsinki, with obtained written informed consent.

### 2.2. Laboratory tests and statistical analysis

The BMI, systolic and diastolic blood pressure, and selected biochemical markers (high-density lipoprotein, low-density lipoprotein, aspartate aminotransferase, alanine aminotransferase, gamma-glutamyl transferase, triglyceride, total cholesterol, glucose, and uric acid) of the participants were recorded. These data were from the case notes and routine laboratory tests of the participants.

The normality of all variables was assessed using the Kolmogorov–Smirnov test. The high-density lipoprotein, low-density lipoprotein, aspartate transaminase, gamma-glutamyl transferase, triglyceride, cholesterol, and glucose were not normally distributed. Subsequently, the Mann–Whitney rank sum test, one-way analysis of variance, and chi-square analysis were used to compare differences of general demographic and clinical data between groups (Table 1). All statistical analyses were performed using the IBM SPSS Statistics software for Windows, Version 20.0 (IBM Corp., Armonk, NY, USA) with a significance level of 0.05 and a two-sided test.

**Table 1:** The demographic and clinical characteristics of AF and IS patients.

Variables	AF group (*n* = 150) (Mean ± SD)	IS group (*n* = 100) (Mean ± SD)	F/Z/*χ*^2^	*p*
Age (years)	65.260 ± 9.825	60.110 ± 9.150	14.832	0.000*
Gender^b^	94/56	71/29	1.857	0.173
BMI	26.118 ± 3.968	25.524 ± 3.173	1.573	0.211
Systole pressure	133.71 ± 23.556	133.39 ± 19.583	0.012	0.912
Diastole pressure	82.20 ± 12.426	84.57 ± 13.233	2.072	0.151
High-density lipoprotein^a^ (mM/L)	1.160 ± 0.466	1.374 ± 2.360	−1.789	0.074
Low-density lipoprotein^a^ (mM/L)	2.549 ± 0.908	2.656 ± 3.023	−1.745	0.081
Aspartate transaminase^a^ (U/L)	29.015 ± 44.518	26.264 ± 8.240	−1.876	0.061
Alanine aminotransferase (U/L)	24.860 ± 20.564	31.132 ± 17.157	2.993	0.086
Gamma-glutamyl transferas^a^ (U/L)	28.120 ± 16.864	39.171 ± 22.779	−2.473	0.013*
Triglyceride^a^ (mM/L)	1.875 ± 1.686	1.269 ± 0.694	−2.007	0.045*
Cholesterol^a^ (mM/L)	4.138 ± 1.215	3.841 ± 1.223	−1.392	0.164
Glucose^a^ (mM/L)	6.642 ± 2.327	5.873 ± 1.831	−2.356	0.018*
Uric acid^a^ (μmol/L)	340.893 ± 97.625	317.623 ± 80.222	1.824	0.180
Hypertension^b^	45/105	46/54	6.635	0.010
Hyperlipidemia^b^	60/90	59/41	8.864	0.003
Hyperglycemia^b^	84/66	73/27	7.422	0.006

All data were reported as mean ± SD or percent using analysis of variance.

^a^ Mann–Whitney rank sum test.

^b^ Chi-square analysis.

* *p* < 0.05.

### 2.3. Data set

The data set consisted of fundus images of the participants with AF and with IS at 548, 605, and 810 nm wavelengths obtained by the fundus multi-spectral imaging system (26, 27).

AF has a fivefold increased risk of stroke (4), and nearly 31% of the patients diagnosed with AF had secondary IS (28). However, approximately 30% of AF patients had no obvious symptoms, and AF is often diagnosed after IS had occurred, especially in middle-aged healthy people, so the patients with IS of unknown etiology are usually considered to have IS caused by asymptomatic paroxysmal AF (7). Consequently, the patients with IS in this study were considered as patients with IS secondary to AF. It has also been reported that some ocular symptoms of neuropsychiatric diseases occur up to 5 years before classical symptoms, and retinal pathological manifestations may precede symptoms of the brain (29). For instance, fundus vascular changes were reported several years before the onset of IS in chronic central diseases, while microvasculature and macrovasculature are affected in an intertwined manner (18). Therefore, it is assumed that the patients with IS have had microscopic pathological manifestations at least 1 year before stroke onset; however, these pathological symptoms cannot be observed by the naked eye, especially in the fundus blood vessels. Furthermore, 61% of the patients with AF were diagnosed with IS within 1 year (4). Therefore, in this study, the IS participants who reported a history of AF or complained of palpitations and arrhythmia were regarded as patients who would suffer from IS within 1 year after AF (defined as the IS group). Since it was difficult to follow up with AF patients about whether they developed IS due to the high dropout rate in outpatient follow-up, the little possibility to predict the exact time of stroke onset in patients with AF within 1 year, and the difficulty to contact patients after their stroke had occurred; consequently, it is a beneficial and advantageous preliminary exploration to regard the fundus images of IS participants who reported a history of AF or complained of palpitations and arrhythmia within the last year as the fundus images of patients who would suffer from IS within 1 year after AF. The AF participants in this study were defined as AF group.

### 2.4. Data preprocessing

**Figure 1:**
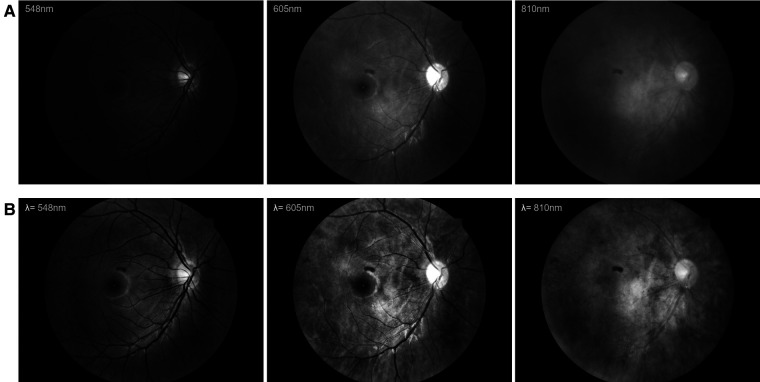
The examples of image pre-processed at different wavelengths. (**A**) The original fundus images. (**B**) The pre-processed fundus images.

The original fundus images were pre-processed according to the following process. First, each image was cropped along the fundus imaging area and normalized to the size of 512 × 512. Second, contrast limited adaptive histogram equalization (CLAHE) was utilized to enhance the content of each spectral fundus image. The illustrated examples of image pre-processed at different wavelengths are shown in Figure 1.

### 2.5. Deep neural network

Three representative DNNs (Inception V3, ResNet50, SE50) were used to validate the detection performance of IS based on multi-spectral fundus images. Specifically, the single-spectral fundus images and the multi-spectral combinations of images at 548, 605, and 810 nm wavelengths were respectively taken as input images to train the models. For multi-spectral combinations, extra convolution kernels were used to extract the desired features. The output value of each mode can be interpreted as an approximate probability of IS occurrence, which ranges from 0 to 1. The decision threshold that predicts IS occurrence based on the model output value was set at 0.5. It took 100 epochs to finish the entire training phase. The training procedure utilized the Adam optimizer with a learning rate of 0.001, a cross-entropy loss function, and a minibatch size of 32.

### 2.6. Performance evaluation

The predictive performance of DNN models was assessed by the receiver operating characteristic (ROC) curves and the area under the curve (AUC) scores of the ROC. Furthermore, the performance was quantitatively evaluated by the accuracy (Acc), sensitivity (Sen), specificity (Spe), positive predictive value (PPV), negative predictive value (NPV), and F1 score. The evaluation metrics were defined as follows:

(1) $$\mathrm{Acc}=\frac{\mathrm{TP}+\mathrm{TN}}{\mathrm{TP}+\mathrm{FP}+\mathrm{TN}+\mathrm{FN}}$$

(2) $$\mathrm{Sen}=\frac{\mathrm{TP}}{\mathrm{TP}+\mathrm{FN}}$$

(3) $$\mathrm{Spe}=\frac{\mathrm{TN}}{\mathrm{FP}+\mathrm{TN}}$$

(5) $$\mathrm{PPV}=\frac{\mathrm{TP}}{\mathrm{TP}+\mathrm{FP}}$$

(6) $$\mathrm{NPV}=\frac{\mathrm{TN}}{\mathrm{TN}+\mathrm{FN}}$$

(7) $$F1\;\mathrm{score}=\frac{2\mathrm{TP}}{2\mathrm{TP}+\mathrm{FP}+\mathrm{FN}}$$

where TP, FP, TN, and FN represent “True Positive,” “False Positive,” “True Negative,” and “False Negative,” respectively.

In addition, we performed global average pooling on the convolutional feature maps before the final output layer (softmax) and used those as features for a fully-connected layer that produces the desired output. Subsequently, the attention map was obtained by projecting back the weights of the output layer onto the convolutional feature maps (30).

### 2.7. Hardware configuration

All DNNs were implemented in PyTorch and trained on an Ubuntu 16.04.12 LST system of x86_64 architecture. The experimental hardware equipment of this system consisted of the Intel Xeon 2.30 GHz CPU with 502 GB RAM and four NVIDIA TITAN RTX GPUs.

## 3. Results

### 3.1. Demographic characteristics

Table 1 shows the demographic and clinical characteristics of AF and IS patients, which showed consistent with the actual characteristics of the two diseases. All the participants had no history of substance abuse or dependence.

### 3.2. The performance of deep network models for predicting the incidence of AF secondary IS

**Figure 2:**
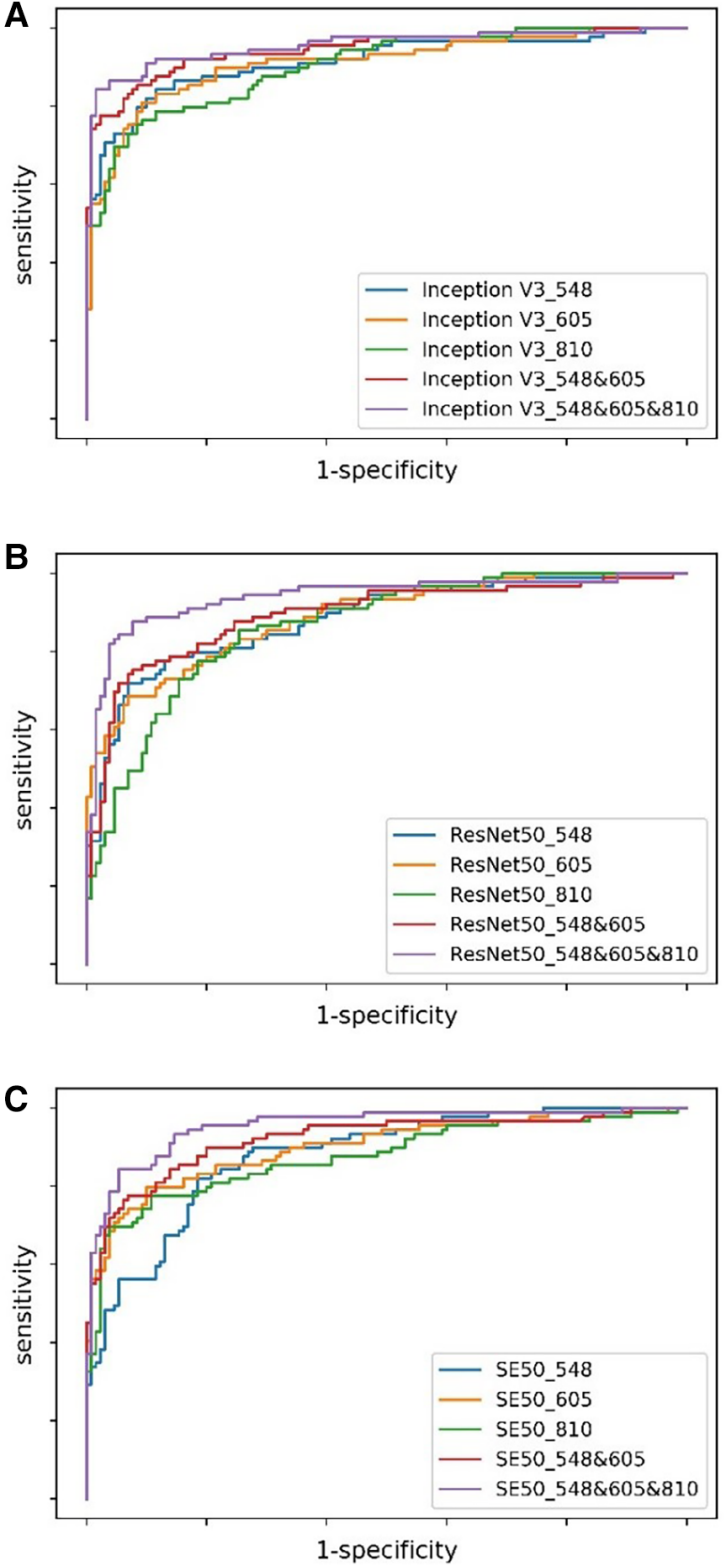
The ROC curves of DNNs using different spectral fundus images as input. (**A**) The ROC curves of Inception V3 with single or multi-spectral fundus images. (**B**) The ROC curves of ResNet50 with single or multi-spectral fundus images. (**C**) The ROC curves of SE50 with single or multi-spectral fundus images.

**Figure 3:**
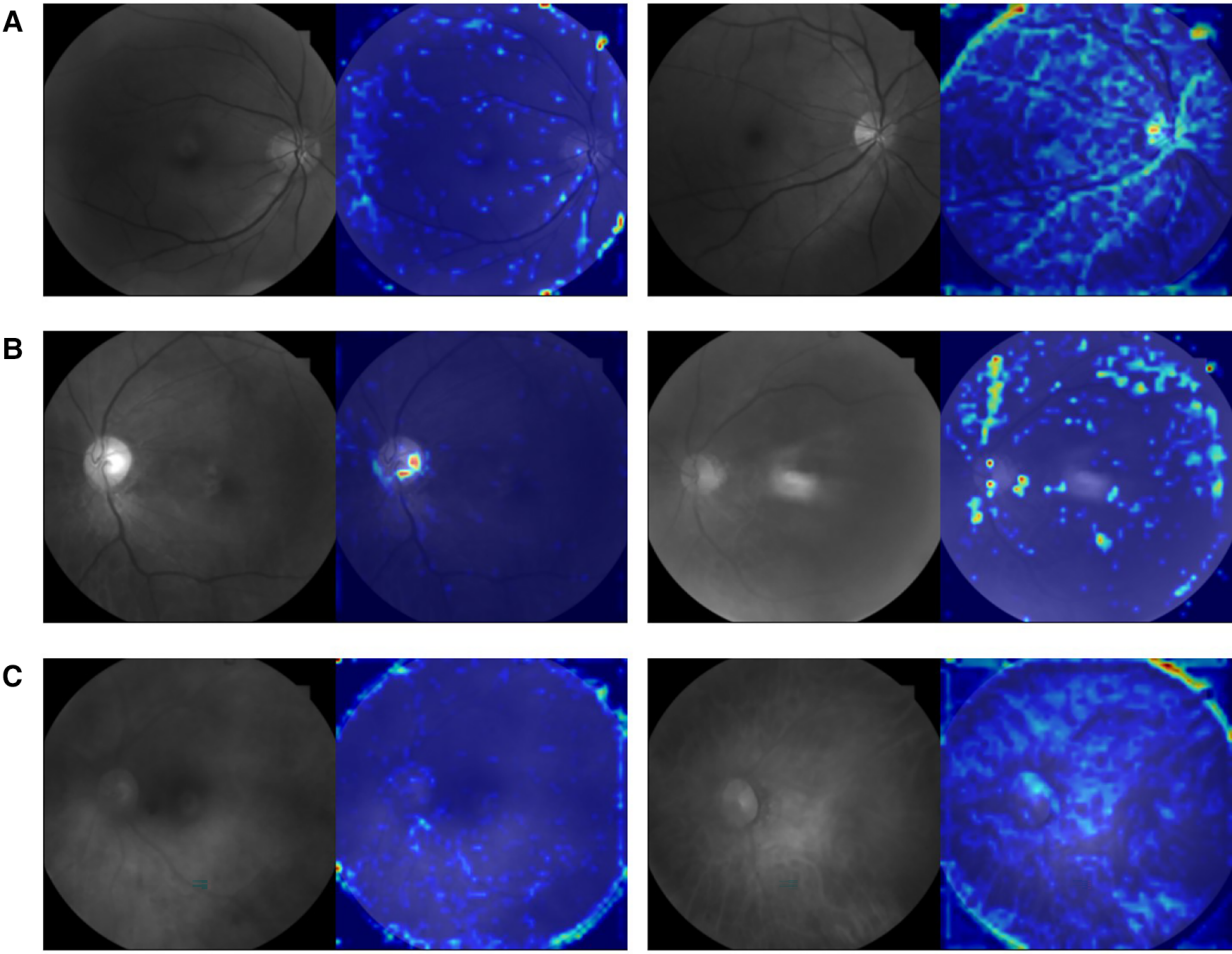
An example picture showing the attention heat map of ResNet50. The highlighted area is the attention distribution used by DNN for prediction. (**A**) The original 548 nm fundus image and its attention heat map of AF group (left) and IS group (right). (**B**) The original 605 nm fundus image and its attention heat map of AF group (left) and IS group (right). (**C**) The original 810 nm fundus image and its attention heat map of AF group (left) and IS group (right).

The evaluation of the three DNN models for the prediction of IS incidence in AF patients are demonstrated in Table 2. The ROC curve is used to evaluate the performance of a binary diagnostic classification method, and its AUC determines the inherent ability of the model to discriminate between groups. Figure 2 shows the predictive performance using ROC curves of different DNNs with different spectral fundus images as input. From the AUC, the multi-spectral classification models had a better prediction performance, especially when all the three wavelengths were used for model training. The attention map could reflect the importance of the image regions. Figure 3 illustrates the heat map of attention, and the IS group had significantly more heat map areas than the AF group.

**Table 2:** Performance of the DNNs trained with different input images at different wavelengths.

DNNs	Wavelengths of input images (nm)	Acc	Sen	Spe	PPV	NPV	F1 score	AUC
Inception V3	548	0.863	0.809	0.900	0.847	0.873	0.828	0.913
	605	0.858	0.753	0.931	0.882	0.846	0.812	0.910
	810	0.845	0.787	0.885	0.824	0.858	0.805	0.904
	548, 605	0.891	0.742	0.992	0.985	0.849	0.846	0.945
	548, 605, 810	0.918	0.843	0.969	0.949	0.900	0.893	0.954
ResNet50	548	0.836	0.719	0.915	0.853	0.826	0.780	0.883
	605	0.826	0.663	0.938	0.881	0.803	0.756	0.887
	810	0.799	0.730	0.846	0.765	0.821	0.747	0.863
	548, 605	0.845	0.685	0.954	0.910	0.816	0.782	0.892
	548, 605, 810	0.904	0.843	0.946	0.915	0.898	0.877	0.940
SE50	548	0.785	0.663	0.869	0.776	0.790	0.715	0.878
	605	0.854	0.798	0.900	0.845	0.867	0.821	0.900
	810	0.840	0.742	0.908	0.846	0.839	0.790	0.875
	548, 605	0.868	0.775	0.931	0.885	0.858	0.826	0.920
	548, 605, 810	0.900	0.843	0.938	0.904	0.897	0.872	0.953

## 4. Discussion

This study demonstrated that the probability of secondary IS in AF patients could be predicted based on multi-spectrum fundus images using deep learning. The accuracy of all DNNs using the single-spectral images or multi-spectral combinations of 548, 605, or 810 nm wavelengths as input reached above 78%, which is better than the logistic regression method reported by Jung et al. (31). The IS detection performance of DNNs using the 605 nm spectral images as input was relatively more stable than using other spectral images. The multi-spectral combination models can acquire an AUC of 0.954, which is at least 0.41 higher than the single-spectral models, suggesting that acquiring different spectral fundus images is advantageous since they might show different pathological microscopic features.

A large portion of the central nervous system is dedicated to vision, and visual problems are prodromal symptoms of IS events, but the treatment for stroke-related vision loss remains limited (32). The non–image-forming or non-visual functions of photoreceptive systems are primarily dependent on melanopsin (33). Melanopsin is expressed by the intrinsically photosensitive retinal ganglion cells (ipRGCs), which are a part of retinal ganglion cells (33). The ipRGCs are the principal conduits for all light input to the non–image-forming visual responses and also receive input from the rod/cone photoreceptors (34), which means that the role of the ipRGCs in non-vision involves the whole photoreceptive system of the eye. The non–image-forming or non-visual responses of the ipRGCs to light include the alignment of the internal clock of the body to the environmental day/night cycle, such as the sleep–wake cycle regulation and the modulation of mood (34). Since the disturbance of sleep circadian has been identified as an independent risk factor for IS (35, 36), this suggests that IS probably related to light through the ipRGCs.

The light was transmitted to the ipRGCs, and they depolarize and project to the suprachiasmatic nucleus (SCN), which further radiates widely to other regions and coordinates the internal circadian synchronization. Disruptions to these biological rhythms can cause abnormal physical changes and diseases including cardiovascular and cerebrovascular diseases (37). For instance, disrupted circadian rhythms are linked to a higher risk of stroke. The disruption of circadian rhythms prior to ischemic events could lead to a prothrombotic state resulting in a heightened predisposition for enhanced stroke damage and poor outcome (38). A recent study reported that chronic circadian disruption increased infarct volume in mice with middle cerebral artery occlusion (39). In human studies, a rotating shift work disrupts circadian rhythms, which is associated with an increased risk of stroke in women with shift work (40). Also, insomnia exacerbates stroke outcomes, including visual impairment (41). Interestingly, it was found that post-stroke patients experienced improved cognitive function and sleep after the 24h naturalistic lighting rehabilitation units (42), certainly pointing to the non-visual effect of light. Post-stroke depression may be related to complex circuitries involving the cortical and subcortical regions (43), and sunlight therapy was reported to improve the mental health of post-stroke patients (42), which was consequently speculated to be related to the ipRGCs signals acting on other brain regions through SCN brain regions.

In the current study, several DNN models to predict the occurrence risk of secondary IS in AF patients based on multi-spectral fundus images all had a good performance, which might attribute to the pathological microscopic changes in the fundus caused by IS itself and its relationship with the circadian rhythm and mood (for example, by the ipRGCs), which are not recognized by the human eye. It is also reasonable to speculate that these long-term, latent, or hidden symptoms in stroke patients may be reflected in the microscopic changes of fundus morphology and then in different spectra. Meanwhile, studies on the relationship between AF, circadian rhythms, and mood are still in their infancy (44). AF is naturally linked to the eye through the vascular system. Some cardiovascular diseases have been found to be related to specific features of the retinal structure and microvessels, suggesting that microvessels and macrovessels are mutually affected in an interwoven manner in heart diseases (18). Consequently, it can be speculated that AF and IS may be presented with different pathological microscopic changes in the fundus.

In addition, the data from this study demonstrated that IS detection performance of DNNs using 605 nm spectral images as input was relatively more stable than using other spectral images, which might be associated with the characteristics of melanopsin or ipRGCs itself. Previously, longer-wavelength photons (∼590–620 nm, including 605 nm, as used in this study) were reported to increase the conductivity of light by triggering chromophore regeneration and increasing the overall intrinsic photosensitivity of the ipRGCs, while shorter-wavelength lights (∼480 nm) favors phototransduction but decreases the overall subsequent intrinsic photosensitivity of the ipRGCs. At intermediate wavelengths near 515 nm (close to 548 nm in this study), the two processes are in equilibrium, which might be embodied by the comfort of green light to the human eye. The orange light (589 nm, close to 605 nm of our study) was consequently demonstrated to activate greater brain activity in several regions of the frontal lobes, which are alertness and cognition, providing strong evidence in favor of a cognitive role for melanopsin (33). The occurrence of stroke-related cognitive impairment has been extensively investigated (45). Neuroanatomical lesions caused by IS on strategic areas such as the hippocampus and white matter might contribute to the pathogenesis of stroke-related cognitive impairment (46). While the 548 nm—and 605 nm—spectral images are often used to calculate blood oxygen saturation, including the fundus multi-spectral imaging system used in this study (26, 27). AF is associated with an increased risk of IS and with post-stroke dementia, which might make its related cognitive impairment common. Although AF without a stroke may also increase the risk for cognitive dysfunction, this is mostly linked to multi-infarct dementia (47). The 810 nm spectral light was mostly used in the low-level laser therapy of transcranial laser therapy as a suitable alternative treatment for stroke (48), and the low-level laser therapy could alter intracellular signaling and change redox states (48, 49). Therefore, in this study, subtle changes in the fundus images at different wavelengths that might not be observed by the human eye or not be presented by mathematical equation could be distinguished by machine learning. This also provides an explanation for the distinguishing of IS from AF by deep learning based on the multi-spectral fundus images. Furthermore, the current data revealed that the multi-spectral classification models had a greater AUC than the single-spectral classification models. Particularly, the best IS prediction performance was obtained when all the three wavelengths were used for model training. The encouraging outcome from the study suggests that biological imaging features of different wavelengths are advantageous to be integrated for IS prediction based on the excellent feature extraction capability of DNNs.

These conclusions can also be reflected in the heat map of attention shown in Figure 3, where the tracks of the attention area all follow the direction of blood vessels, and the IS group had significantly more heat map areas than the AF group. Although the model will inevitably allocate its attention to noisy or high-frequency areas (such as edges) due to the limitations of data volume and the impact of image noise, our model still exhibits different attention distributions in the IS and AF groups, supporting our conclusion. In Figures 3A,B, the trace of the heat map region is more obviously distributed along the blood vessels, which is consistent with the light characteristics of 548 and 605 nm spectral images and their use in the calculation of blood oxygen saturation (50). Figure 3C shows a divergent trend, which is consistent with the view that stroke may be a chronic inflammation of the global brain (51) and is similar to the reason why 810 nm light can be used to treat stroke with transcranial laser. Nevertheless, that has to be verified by more samples.

There are some limitations to this study. First, there was a difference between the age of participants in the AF and IS group, and the age of the AF group was older than that of the IS group, which might be seen as a confounder when interpreting the results. Second, a larger number of participants might further support the results of this study. Finally, although there are no validation sets in this study, as a prospective and exploratory study, it is considered to provide a new vision and thought for the research of chronic cardiovascular diseases such as AF. We will also continue to collect samples to further verify our conclusions.

## 5. Conclusion

The probability of secondary IS occurring in AF patients could be predicted based on multi-spectrum fundus images using deep learning, and combinations of multi-spectrum images improved the prediction performance of DNNs. Acquiring different spectral fundus images is advantageous since they might show different pathological microscopic features. Considering the convenience and non-invasiveness of measuring the eye, this will provide a new clue for the early prevention of cardiovascular and cerebrovascular diseases. More importantly, the method in this study is a beneficial preliminary and initiative exploration for diseases that are difficult to predict the onset time, such as IS.

## Data Availability

The original contributions presented in the study are included in the article/Supplementary Material, further inquiries can be directed to the corresponding authors.
